# Psychology Students' Perceptions of COVID-19 in a Death Education Course

**DOI:** 10.3389/fpubh.2021.625756

**Published:** 2021-04-16

**Authors:** Ines Testoni, Erika Iacona, Cecilia Corso, Sara Pompele, Laura Dal Corso, Hod Orkibi, Michael Alexander Wieser

**Affiliations:** ^1^Department of Philosophy, Sociology, Education and Applied Psychology (FISPPA), University of Padova, Padova, Italy; ^2^Emili Sagol Creative Arts Therapies Research Center, Faculty of Social Welfare and Health Sciences, University of Haifa, Haifa, Israel; ^3^Department of Psychology, University of Klagenfurt, Klagenfurt, Austria

**Keywords:** COVID-19, death, death education, psychology students, lockdown experience

## Abstract

The systematic removal of death from social life in the West has exposed people living in areas affected by COVID-19 to the risk of being unable to adequately manage the anxiety caused by mortality salience. Death education is a type of intervention that helps people manage their fear of death by offering them effective strategies to deal with loss and anxiety. To that end, a path of death education has been carried out with University students of psychology. The main purpose of the research is to understand how students who participated in the death education course perceive the lockdown experience in light of course teachings. The research was carried out at a University in northern Italy in an area severely affected by COVID-19, during the first year of the pandemic. The group of participants included 38 students, 30 women and 8 men, with an average age of 25.45 years (*SD* = 7). At the end of the course, the students could respond on an optional basis to the request to comment on the training experience according to what they experienced during the pandemic. A thematic analysis was subsequently carried out on the texts, which made it possible to identify the most relevant thematic areas for the students. The qualitative analyses permitted recognition of three main forms of discovery: the removal of death in contemporary culture; the importance of community, ritual and funeral, and spirituality; and the significance of death education for future health professionals. The texts have highlighted how the removal of these issues exposes people to the risk of being unable to handle extremely painful events such as those related to dying. The results show the positivity of death education pathways conducted at the University level to help students reflect on these issues and manage the related anguish.

## Introduction

During the early months of 2020, the Italian health system was forced to grapple with the recent pandemic caused by the spread of COVID-19 and the subsequent sudden increase in death rates, which constituted a huge, and, at least in the last decades, unprecedented, public health concern, and challenge. The national health emergency significantly affected life and work, and the restrictive solutions negatively influenced almost all social sectors, including those of the educative area (primary and secondary school, and universities).

A theory that could help understand the significant negative psychological impact of COVID-19 on society is terror management theory (TMT), which states how the constant conflict between survival instinct and the awareness that everyone must die sooner or later (called mortality salience) causes intense cognitive dissonance and sufferance to people, who therefore constantly attempt to reduce mortality salience itself (1). Terror management theory focuses therefore on the crucial role death awareness plays in human life. Over the past 35 years, hundreds of empirical studies have confirmed how diverse aspects of human behavior are directly influenced by this, showing the role of proximal defenses, which are cultural constructions that enable people to think of themselves as valuable contributors to a meaningful, significant, and permanent universe, and distal defenses, which help individuals to give sense to the relationships between life and death (2).

It is therefore understandable how in a period such as the present one, with a world pandemic of highly contagious nature that is causing an enormous death toll, how mortality salience could become almost impossible to hide, and how the virus plays very important roles in spawning anxiety that could result in critical behaviors and situations. From the TMT point of view, therefore, mortality salience caused by the pandemic plays a central role in driving the attitudes and behaviors of most of the population in each country plagued by the virus (3–5).

The systematic removal of the reflection on death and dying that has particularly characterized Western culture in recent decades (6) has left individuals unprepared in the face of the massive amount of news that activates the mortality salience in the pandemic period. In recent years some death education pathways have been developed to help people, especially adolescents, to acquire familiarity with the idea of death, feeling free to discuss it with peers and expressing the related feelings and thoughts, especially through the mean of the arts, which allow them to be as free as possible in their elaboration of the concept of death and dying (7). For example, an approach that is proving to be extremely useful is psychodrama, through which people, usually divided in groups, have the possibility to represent and give life to both past experiences and personal fears or fantasies through the mean of dramatic enactment and which is easy to implement with adolescents in a context of death education, during which they can live again and possibly change, in the protected psychodramatic stage, past griefs, or even represent their personal idea of death, and share it with the rest of the group, discussing it together (8). A very common psychodramatic techniques applied to death education is for example the “empty chair,” in which participants are sitting in front of a physical empty chair on which they imagine a deceased loved person or even death itself as a personified character is sitting, thus allowing them to elaborate their feelings and thoughts concerning the theme of dying in a dialog with the person/entity on the empty chair (7).

Another methodology that has proven to be very useful in death education courses with groups of adolescents is for example photovoice, in which they are asked to produce pictures concerning the theme of death and dying and how they personally perceive and represent it, and later show them to their group and discuss them with the others (9). These experiences seem to have demonstrated that consciously managing the issue of death and the negative feelings associated with it can indeed strengthen people's resilience and allow them to feel less frightened by it (7, 10). Death education aims therefore to promote dialogue and reflection on issues that are usually removed from daily dialogue because they prompt anxiety and sadness (11). The greater awareness of the terror of death and the resulting defensive dynamics help people face the difficulties that arise from mortal situations and help others relate to people who are suffering from a personal loss (12). Moreover, literature has also shown that reflecting on death and human vulnerability can also help to reduce anxiety and to better manage information related to the preservation of one's health (13). For example, a qualitative study of community death education in which participants were offered a death education course and were later asked to imagine they were affected by Amyotrophic Lateral Sclerosis (ALS) and had to prepare their Advanced Treatment Directives (ATDs) highlighted how reflecting upon the themes of the knowledge of having to die, palliative care and ATDs significantly helped participants to think to their death in a less distressful way and to be able to plan their future healthcare treatments and fundamental desires with less anxiety (14). Death education has moreover been demonstrated to be useful also to help prevent other significant public health concerns, such as for example the issue of suicide risk, especially among adolescents (15) and the likeliness of smoking (16).

The strength of these paths lies in the reflection on transcendence and spirituality, which find in distal defenses their power to reduce anxiety and fear (7).

It is reasonable to believe therefore that death education courses could indeed provide an important support also in dealing with the current pandemic situation, the present most significant and dangerous worldly public health crisis.

As stated in *The Lancet* (2020): “A pandemic is a cause and powerful amplifier of suffering, through physical illness and death, through stresses and anxieties, and through financial and social instability. Alleviation of that suffering, in all its forms, needs to be a key part of the response” (17). Such an extreme negative impact the COVID-19 pandemic has and the mortality salience it elicits, imply that the contrast to the pandemic is also carried out with initiatives of consciousness-raising that make people aware of their psychological frailty in facing death. These further undesirable effects worsen the state of psychosocial distress caused by the virus. Recent studies show the usefulness of conducting death education courses with children, adolescents, and University students. If conducted properly, it is possible to manage in a positive way the effects of the path of reflection on death, on the fears it arouses, and on the effects (9, 15, 18). Therefore, it could help in critical periods of the pandemic to set up special paths of death education that support students in becoming aware of what happens and their experiences in this regard (19).

In Italy, Law 38/2010 on “Provisions to ensure access to palliative care and pain therapy” (8, 20) has included in all University courses of medicine, nursing, psychology, and social services that must address the issue of death to enable future health professionals to acquire basic skills related to palliative care (8). In one of these courses held during the first phase of the pandemic in Italy, in one of the geographical areas most severely affected by the infection, the psychological effect of the COVID-19 experience was explicitly addressed. This intervention coincided with the lockdown period imposed by the Italian government since March 2020 (21), and for this reason the lessons were held at a distance. The literature on educational pathways during COVID-19 focused especially on internal and external changes in the formative paths that institutes, teachers, and professors had to make to rapidly adjust their modes of teaching (22, 23). Since there is not yet much literature on specific death education activities useful to manage anxiety and fear of death, this study presents the results of a qualitative survey with students who participated in a course for palliative care where they could face their emotions and feelings related to the pandemic.

The course was realized with undergraduate psychology students at a University in northern Italy, where the pandemic effects were particularly severe, and it focused on palliative care, including death education issues with particular attention paid to the current COVID-19 pandemic.

The present research, instead, aimed to understand how the students who took part in the teaching experienced the lockdown period in light of the preparation that the course provided them. We wanted to investigate how dealing with death education issues influenced the way the participants lived and perceived quarantine and the constant mortality salience caused by the daily information. In particular, we tried to recognize and outline the students' personal feelings with respect to the death education course and its effect, extrapolating the fundamental pivots on which they anchored the capability to think of and talk about issues relating to death and dying as proposed in the course.

## Methods

### Participants

The research involved 38 students, 30 women and 8 men. The average age was about 25.45 years (*SD* = 7). The participants were all psychology graduates who were pursuing master's degrees in psychology. In order to recruit them, researchers presented the study protocol and aims in detail during one of the first online lessons of the palliative care and death education course. During the occasion, all the necessary information concerning the protection of participants' personal data and confidentiality, as well as concerning the possibility to choose freely whether to complete the research procedure or withdraw from it at any time without having to give any explanation for it and without risking any penalty of any kind, were given too, and students were encouraged to ask all the questions they needed. The researchers also made clear that taking or not taking part in the study would not have affected in any way students' final score on the exam at the end of the course, in order to ensure that the participants' motivation for joining the study was of simply curiosity and desire to aid psychological research and not linked to fear of repercussions, if they chose not to participate, or rather the need to obtain some benefits if they did take part in the research. The fact that their answers would have been used anonymously for the research results was also specified, in order to prevent as much as possible any form of social desirability in their given answers. All this information were repeated to those who actually decided to participate and presented in a written informed consent they were asked to sign before starting with the concrete study procedure.

The research followed the APA Ethical Principles of Psychologists and Code of Conduct and the principles of the Declaration of Helsinki, and approval was obtained from the Padua University Ethics Committee for Experimentation (n. 57BC2002FDF5CBD4292F5F86AA077F23). The names reported below are fictious. When necessary, the quotation has been camouflaged to avoid revealing a participant's identity.

### Data Collection

This study followed a qualitative research design (24) within the grounded method (25), considered in the literature to constitute the most reliable methodology for investigating issues pertaining to health problems that are not yet considered (26). This methodology can also generate reflections to a much greater extent than the classical technique of direct interview or questionnaire (27).

Participants, as has been previously mentioned, attended a death education course which focused on palliative care, including death education issues with particular attention paid to the current COVID-19 pandemic. It was structured in frontal lectures delivered in telematic mode through video lectures and in group work. In addition, interventions were proposed by experts in the field and peer group work to allow students to better understand some nodes related to the psychological effects of the death experience and to allow them to confront each other. The aim of the course was to provide knowledge to acquire awareness about the management of terror of death and dying, loss, grief, and mourning, considering such themes from different points of view and involving psychological but also sociological, philosophical, and religious aspects and theories. The course, which is implemented every year for psychology students, usually also dedicates some time to the related themes of euthanasia, assisted suicide, and related bioethical issues, and to the aspect of psychological and medical support to the elders. However, because of the intense impact COVID-19 and the perceived urgent need students had to reflect upon the pandemic psychological and social implications, in the current version of the course these themes, even though fundamental, inevitably had to take second place, and were therefore less considered and discussed with students. With respect instead to the management of the COVID-19 experience, the main objectives of the course were: becoming aware of the forms through which death anxiety characterizes human suffering; knowing and understanding how terror of death influences human behavior; and considering the current situation in light of the death studies discussed.

Approximately 3 weeks after completion of the course, which was considered the proper amount of time to allow them to elaborate the personal experience lived during the course itself, participants were asked to write an essay concerning what they had understood about their experience of the pandemic and their awareness with respect to the effect of the widespread and increasing mortality salience. The specific setting for the procedure was represented by each participants' home, since at the time of the implementation of the research, right after the course, universities were still closed, and only online lessons were allowed, to comply with the COVID-19 regulations on social distancing. The participants were given an hour and a half to complete the assignment, and they were asked to send the written reports to the researchers through their e-mail.

### Data Analysis

The written texts obtained constituted the material for the qualitative research. The corpora obtained by the students' texts were analyzed following rigorously thematic analysis steps: reading, tracing the units of meaning, examining the redundancies and differences, reflecting on the units of meaning to extrapolate the theme being transformed into scientific language, and finally, formulating a consistent description of personal experiences (28, 29). The study identified the most salient thematic areas that the students involved had highlighted with their responses, through the recognition of the most relevant categories that facilitated a detailed conceptual analysis of their perspective (30). The paper-and-pencil analysis operations were then integrated using the computer program qualitative analysis software Atlas.ti, which has been precisely designed to aid researchers in qualitative data interpretation, allowing an analysis that is as objective as possible. Moreover, in order to ensure the findings would have been interpreted in an unbiased way, two of the authors have independently and simultaneously proceeded with the data analysis, and they have subsequently compared each other results, in order to check the eventual presence of high discrepancies between each other findings and to understand why this could have happened. In this study specific case however, no peculiar discrepancies have emerged and both researchers reached very similar conclusions.

## Results

Thirty-eight texts were collected for a total corpus of 16,454 words. The texts average 433 words each. A thematic analysis was conducted to highlight the thematic areas present in the corpus. Three main thematic areas have been identified: 1—Removal of death in contemporary culture; 2—The Importance of Community, Ritual and Funeral, and Spirituality; 3—The Significance of Death Education for Future Health Professionals.

Concerning the first thematic area, “Removal of death in contemporary culture,” some of the fundamental elements that have emerged are:

—Students becoming aware of the social strategies that characterize the removal of death from the consciousness of everyday life;
—The importance of reflecting on fundamental existential themes, which in turn developed into the importance of acquiring broader categories of thought and a suitable language to give meaning to life and the pain of loss.

Concerning the second thematic area, “The importance of Community, Ritual and Funeral, and Spirituality,” the main themes that have emerged are:

—Understanding the suffering of those who had to deal with the loss of a loved one during the pandemic without being able to accompany him or her and without being able to celebrate an appropriate funeral ritual;
—Reflection on the importance of the spiritual dimension, regardless of personal attitudes toward religion;
—The need for closeness with others and sharing the negative feelings caused by the pandemic.

Lastly, concerning the third thematic area, “The Significance of Death Education for Future Health Professionals,” the fundamental elements that have emerged are:

—Acquisition of awareness and personal reflection, especially concerning the recognition and control of the negative emotions that occur in situations related to death;
—Better management of the social trauma caused by COVID-19 thanks of the Death Education course;
—The possibility to refocus on life instead of the fear of death;
—Recognition of the need for adequate preparation regarding the topics covered by the course.

**Figure 1:**
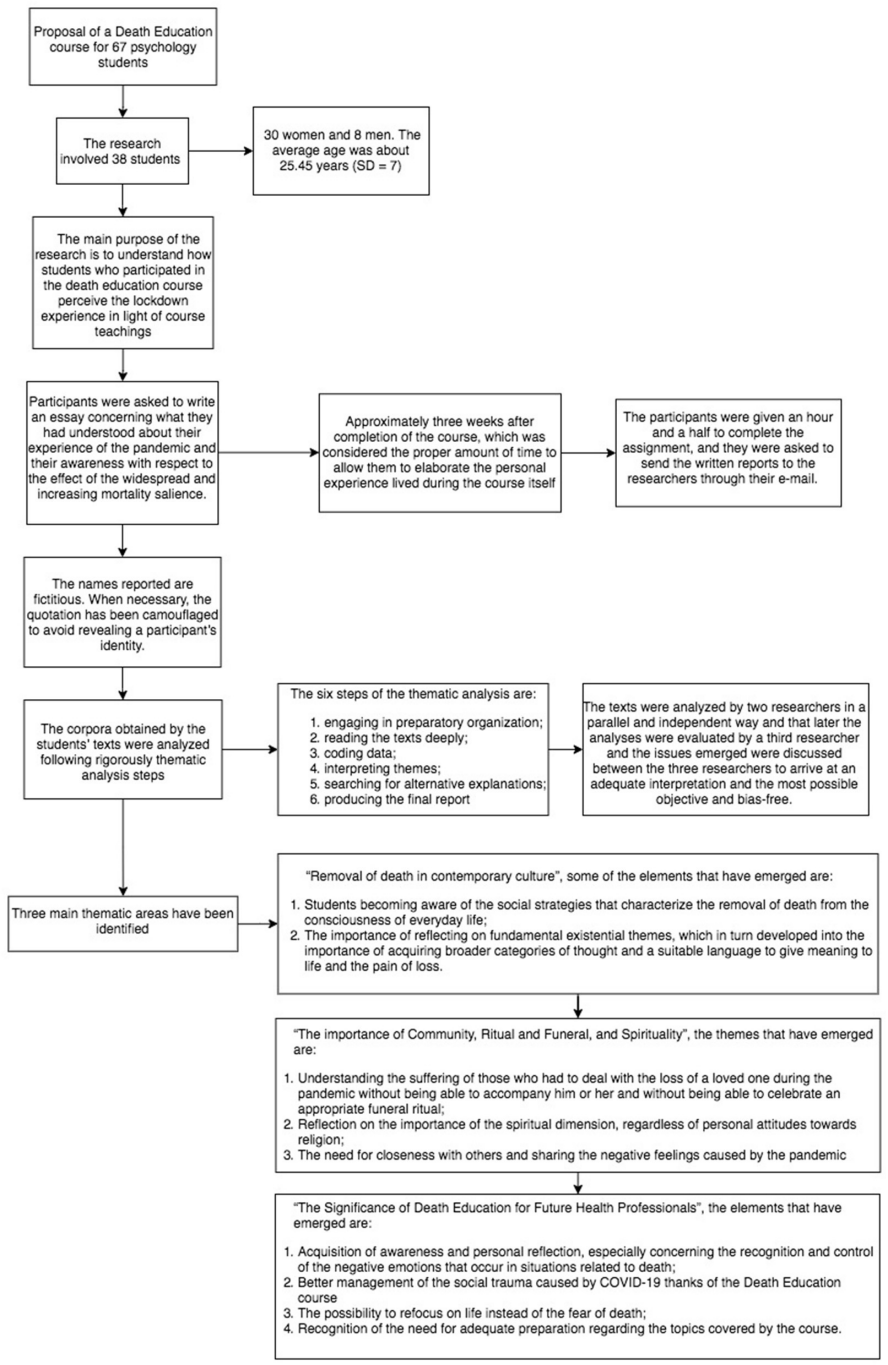
Flowchart describing the phases of the study.

In order to visually summarize the present fundamental thematic areas and their main sub-themes, a figure (Figure 1) has been created that illustrates the results findings. In the following section of the manuscript, each thematic area will be presented in depth, and quotations from the very narrations offered by participants will be presented.

### First Prevalent Thematic Area: Removal of Death in Contemporary Culture

More than half of the participants acknowledged that they had reflected for the first time on the fact that death is a real cultural taboo, that it is not at all a socially accepted topic that can be freely spoken about. Valeria (28 years old) expressed for example how during the period of the pandemic, according to her, people have been forced to deal with the concept of human vulnerability and death, on a daily basis: “Suddenly, during the course, I became aware of how I had never had the chance to reflect on the meaning of death and how I could never talk about it with anyone. During the lockdown, instead, we were all forced to reckon with this censorship, because suddenly there was no other talk about it in the media, but of course everything always referred only to COVID-19. We all realized that we were absolutely unprepared for this sudden forwarding of messages related to death and this inevitably distressed us. I for one always believed that there was no point in thinking about death […].” Unlike Valeria, who expressed how before the course she had never really thought about death, Sofia (23 years old), for example reported instead she had already felt the desire to explore the theme in her life, even though it also scared her, but that was never possible and she did not know how to face this: “When I was a child and a teenager, I often felt the need to talk about it, to confront it, to tell my family how terrified I was at the idea that something might happen to them. But it has never been possible to address this issue. I wondered what meaning life would have had if my parents had died, […]. I wondered what the relationship between the meaning of life and the attachment I felt for my loved ones was.”

Federico (32 years old), also expressed how the context of the pandemic made his fear of losing his parents and to die himself, even stronger, and how the course on the contrary helped him find a way to deal with this fear: “I was very scared of losing my parents and of dying myself too, however the course helped me, especially analyzing other people's different representations of death, discussing about annihilation and nothingness and especially the thought of the Italian philosopher Severino.”

Alessandra (28 years old) reported as well how the pandemic has allowed her to realize how much of a taboo the theme of death is still considered: “I was surprised to realize that we live in a delusional delirium that makes us believe that we can avoid considering arguments concerning death in everyday life. I suddenly realized that we procrastinate the moment of encountering this topic for so long, that when it is necessary to deal with it we are unprepared. This is exactly what is happening now, that we are forced to deal with the anguish of death without having tools.”

As evidence of what Alessandra said, Gioele (23 years old) tested in his real life the fact that people avoid talking about death and dying: “It was useful and interesting because within my group of friends, now that we have had the opportunity to meet again, we have dealt with the topic of Covid and the topic of death and I was able to feel the block we have also from a practical point of view. When we have to talk about death.”

Similarly, Giulia (23 years old), expressed as well how the course helped her understand the importance of actively talking about death: “Understanding the importance of talking about death has given me the opportunity to experience firsthand how beneficial a constructive dialogue on death and fear of death can be. Perhaps the current pandemic could be managed in a different, less distressing way.” Ivan (25 years old) recognized too the help given by the course, and referred it directly to his personal experience, saying: “I also noticed the decrease in my tendency to change the subject when someone communicated to me a grieving situation.” In line with these considerations, Roberta (23 years old) concluded by saying that she hoped the experience of the pandemic would not lead to a new community removal of the concept of dying: “now the important thing to do is to make sure that compared to this terrible experience of the COVID-19 there is no sort of mass social removal. It would be important to find a way to process it in a meaningful way. Indeed, I have suddenly understood that it is not with the removal and avoidance of themes that remind us of our finitude, that our impact on death is avoided.”

Rosa (31 years old), reported a thought very similar to Roberta's one, by saying that what the course really left her was the awareness that death is actually part of life, it is natural, and should not therefore be demonized or avoided as a theme: “I believe this is what I understood thanks to this course that I attended exactly during the pandemic: the awareness that death is a part of life, that it can happen even though we do not expect it, and that we should learn to face it serenely and to accept it, even though fearing it is unfortunately part of the social and cultural background we live in.”

Similarly, Alberto (24 years old) reflected on our society and how difficult it is for it to relate with the theme of death: “This path, through the pandemic, has allowed me to reflect on the relationship between death and Western society, on how our society is not very accustomed and not very close to what is most intimate and natural the human being possesses, that is the awareness of our own finitude.”

A slightly different point of view on the issue was brought by Alice (24 years old), who instead considered the professional aspect and the importance to be able to manage, as a healthcare professional, the theme of death with patients without feeling overwhelmed: “I realized the importance of beginning to speak honestly about death, how much confusion there is around these issues, both in terms of unconscious beliefs and representations, and in terms of misinformation. It is even clearer to me the importance of good information, especially if you work with those who are sick, with those who are ill.”

### Second Prevalent Thematic Area: The Importance of Community, Ritual and Funeral, and Spirituality

Significantly linked with the first thematic area, the second one highlighted a further discovery, that is, the importance of the accompanying rite and funeral of those who die and of the community. Indeed, the very condition of constant social and cultural removal of death and its devastating effects during a period of high mortality salience as the pandemic one brought participants to acknowledge the vital psychological and social function funeral rites possess, since they help mourners to feel embraced by their community and better elaborate their grief. This was expressed by many participants, for example by Luisa (26 years old): “I thought about those who were sick in the hospital. I felt a great relief in being able to share my fear with others, through the internet, and this allowed me to feel less alone.” Similarly, Maria, a woman of 65 years old, the only participant who had a significantly different age from the rest of the group, highlighted how during the lockdown she felt it was fundamental for her to be resilient and maintain a contact, even though not physical, with her loved ones, and how the course helped her realize how fundamental social relationships are and how important it is to share feelings and fears with others especially in such periods of global crisis: “I especially felt I had to nurture my resilience in that moment by regularly having telephone and on-line contacts with my friends and relatives since I felt I could share the same experience with them. And preparing this exam has helped me in this purpose, I could take care of my loved ones even though at a distance, have authentic and supportive relationships, give voice to my fears and share them.”

Giada (23 years old) proceeded in this direction as well and specified she felt the need to live even more intensely when she thought about those who were left alone to suffer: “During this lockdown, I committed myself to live more intensely the relationship with my parents, to grow together. I looked for more moments in which we could communicate, and I thought about those who had to live the suffering alone.” Costanza (23 years old) expressed the same thoughts as well: “Thinking about those who die in the hospital alone, I understood how important it is for a community that pays attention to the pain caused by death. A community is truly such if it never makes you feel alone and creates a network of support and sharing ready to help and support you in your time of need. I had never considered this important aspect of social life before.” Monica (23 years old) highlighted the importance of a support network as well especially in such a delicate phase as that of the end of life: “Communication and sharing on a collective and community level of suffering are the key to improve grief processing and support people who have and are suffering now. I didn't think about this before.”

These considerations on the importance of sharing and emotional support led some participants to also consider the fundamental role psychological help can have in painful and complex situations as the present one. Luca (23 years old), for example, said: “I have therefore realized how important is the profession that I hope to be able to perform in the psychological field, I thought about the support that can be given and how fundamental it is to receive it especially in situations like the one we are living.” Agreeing with him, Lucia (23 years old) said: “Before this course, I had never grasped the positive aspect of being able to be next to a dying person. […] Before, I had never thought about the importance of the human aspect of health care work. […] Instead, understanding this, relationships become essential tools.”

According to the participants, the importance of relationships is more evident in moments of difficulty, such as the pandemic, and the support of the community becomes crucial in the moment of mourning when its members gather around the mourners, as Costanza said: “I was struck by the difference between grief and mourning and the passage from the first to the second, thanks to the funeral rite and the closeness of the community. I understood that they are fundamental elements that, if they are missing, important problems can arise. For this reason, I believe that we should support those who have seen their loved ones go in an ambulance to the hospital and have not seen them come back.”

But the funeral ritual is not meant to help only the suffering people to mourn. Vanessa (24 years old) commented on the function of the funeral from the point of view of collective support as well: “Thanks to this course, I understood the importance of the funeral ritual and the closeness and support of the community at that time to the mourners. I had never asked myself the question, or rather, it seemed unimportant. Now I finally understood the importance and meaning of this celebration, even though I am not a believer.” Agreeing with her, Lorenzo (24 years old) said “I think that the course has helped me above all to give words to something that I already only sensed, that is the value of the rituality of death. Seeing how this had been made impossible by the rules on social distancing, and the anguish, dismay and anger that this caused in the population was like re-meaning the value of this experience.”

Lastly on this thematic area, Roberta also highlighted another fundamental aspect, that is, the difference between religiosity and spirituality, which in turn allowed her to feel freer to explore the latter in her personal dimension: “Understanding that the spiritual dimension is different from religiosity and what revolves around religion has finally allowed me to open a dialogue with myself to reflect deeply on the coordinates of life. Being able to do this now, in this time of pandemic was important to me.”

### Third Prevalent Thematic Area: The Significance of Death Education for Future Health Professionals

The awareness of having to realistically reckon with the limit and in particular with the limit of life, put in the foreground by the pandemic, as well as the paralyzing lack of proper rites to support mourners in those moments, allowed the participants to also recognize in turn the positive aspects of sharing thoughts, opinions, and feelings within educational paths that allow to place death in a historical-cultural context, especially in moments of extreme uncertainly as the present COVID-19-related one. Participants indeed expressed that they believed proper death education paths could represent a valuable tool to strengthen people's psychological resources and help them face these complex situations. For example, according to Alberto: “Looking at death by tracing a historical, symbolic-cultural and scientific-psychological path has allowed me to acquire a greater awareness and a greater critical sense about the events that, especially during the acute phase of the pandemic, congested our windows and the media.” More specifically, the course of death education has helped some participants to better understand and formulate their own way of understanding death, as for example Chiara (23 years old) expressed: “The study of themes related to death and dying has given me the opportunity to better understand how death has been understood during the various eras and in this way to become more aware of how I understand death, what value I attribute to it, giving me the opportunity to reflect on how I relate to the idea of death, especially sudden death. It seems to me that this helps me to better manage what we are living.” Valeria seemed to echo: “This course has given me the tools to overcome that step of anguish that makes death fearful and unthinkable and has taught me to look at the theme in its entirety, without taking anything for granted.”

Relating to this, Luigi (24 years old) expressed as well how the course helped him understand the pandemic situation and live it with more maturity: “I do not know if I am more serene than it would normally have been, but certainly opening my eyes from a scientific point of view on the topics covered in the course was the basis for reading this situation with maturity and awareness.”

According to Lucia, during the course she was finally able to face the fear to talk about death she had always had: “Precisely in this period, I have given shape to this fear of talking about death, I was able to understand its origin and the great shortcomings that there are in our society.” According to the participants, being able to understand topics of this magnitude allows us to treat them from a more objective point of view and with less emotional involvement, as for example Elisa (23 years old) explained: “What helped me most was hearing about death with a critical eye and a scholar helped me to make the topic more normal and more treatable. This fact has allowed me to distance myself from the fear that death inevitably implies.” The positivity of the course was also linked to the fact that it allowed to focus on life and to recognize the importance it deserves. Dealing with these issues, as Manuela (23 years old) said, makes it possible “to give more relevance to the meaning and significance of lived existence rather than the fear of the finitude of life,” while in a very similar way Federica (27 years old) expressed herself: “Talking about death gives meaning to life, makes you feel more alive. This course has allowed me to face the lockdown by valuing every moment.”

## Discussion

**Figure 2:**
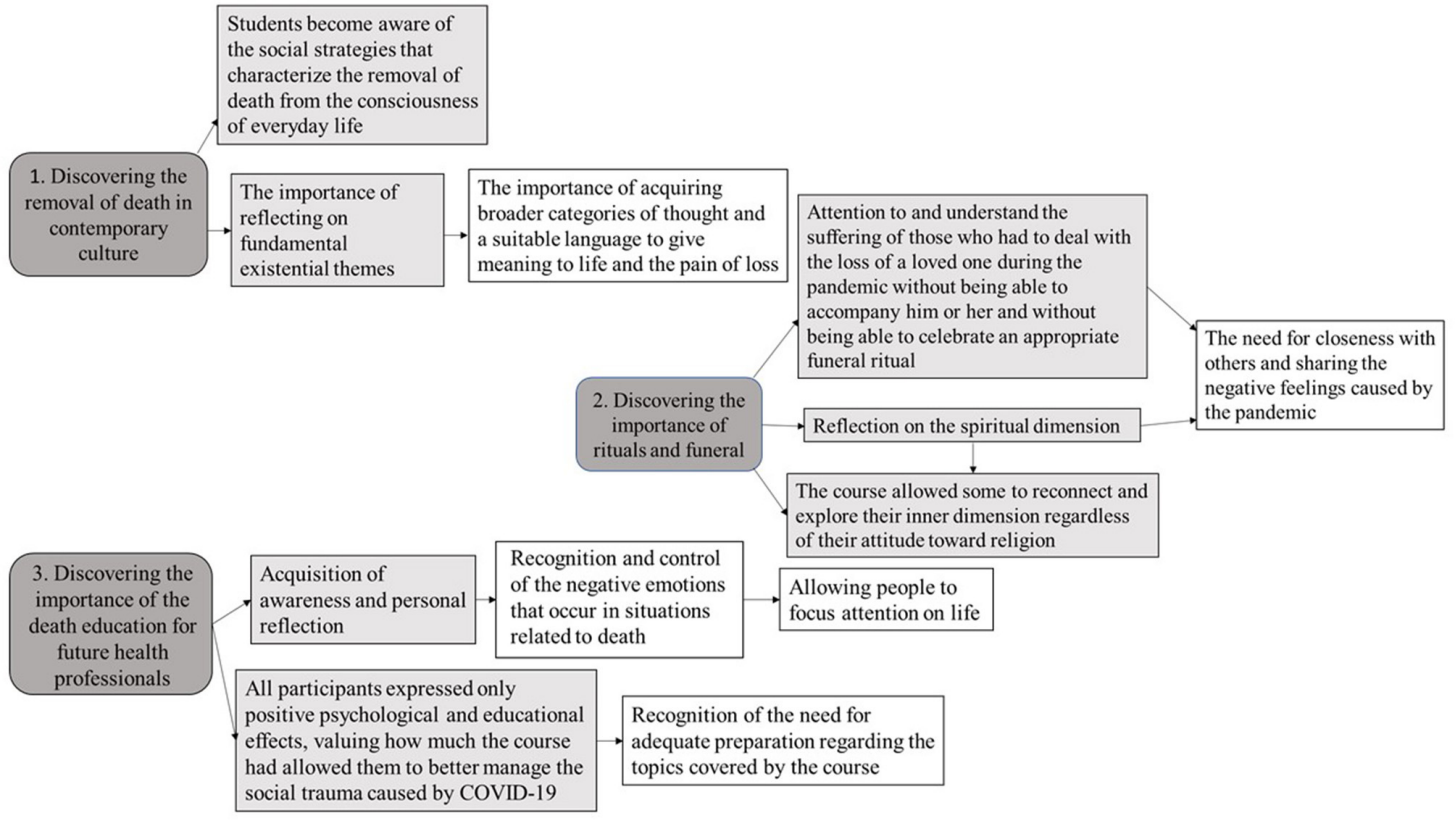
Findings of qualitative data analysis. Main themes and their relationships.

The present study, which has been summarized from its elaboration to its main results in Figure 2, aimed to explore psychology students' experiences and perspectives concerning the present COVID-19-related mortality salience situation and especially lockdown measures, in light of the death education course they attended. Concerning the theme of death and the end-of-life, international studies have indeed already highlighted how other healthcare students, especially medical and nursing ones, report not to feel adequately prepared to face these issues and support a dying patient, and how the intense need for proper death education and palliative care trainings is indeed still extremely high (31, 32).

The current COVID-19 pandemic has exacerbated this critical issue, causing intense feelings of uncertainty and high distress among healthcare students, who had to face, generally unprepared, an unprecedented sanitary crisis with extremely high deathrates (33, 34).

The present research findings have highlighted these critical aspects as well in relation to psychology students, concerning whom less studies have been conducted, who had to live and learn in the COVID-19 period, and who had to deal with a related abrupt increase in mortality salience, while attending a death education course. From the obtained results, three main forms of discovering that characterized students' feeling, and learning have emerged: first, the discovering of the removal of death in contemporary culture; second, of the importance of community, of rituals, and of funeral, but also of spirituality; and third, of the significance of the death education for future health professionals.

With respect to the first thematic area, the most important feeling students experienced was to become aware of the social strategies that characterize the removal of death from the consciousness of everyday life, realizing how this has led to a general inability to deal with the shocking experience of the pandemic. This paralyzing lack of adequate skills related to death and the end-of-life, exacerbated, and made even more evident by the pandemic, has already been reported by other healthcare students' as well in literature, especially undergraduate nurses and doctors, as has already been highlighted, who have reported how overwhelmingly unprepared they felt concerning the present sanitary emergency (35, 36). We think that the study of the TMT contents helped participants to develop a broader perspective of this problem (4). Some students also reported personal experiences, very specific ones that we omitted in this report, because they could make the participants recognizable. These testimonies described important losses and unanswered requests and needs for support. Supporting literature addressed the importance of reflecting on fundamental existential themes for healthcare students in order to acquire broader categories of thought and a suitable language to give meaning to life and the pain of loss and acquire the adequate professional skills to be able to deal with these themes in their future job (37).

The second thematic area highlighted a further discovery, significantly related to the first one, that is, the importance of the accompanying rites of those who die and funerals. This has made it possible to pay attention to and understand the suffering of those who had to deal with the loss of a loved one during the pandemic without being able to accompany him or her and without being able to celebrate an appropriate funeral ritual, and this is in line with other literature findings, which have highlighted how healthcare professionals, students, and trainees in general felt the painful burden of having to assist and support patients who were indeed dying alone and to support family members who could not properly accompany and later mourn their loved one (38, 39). In this scenario, reflection on the spiritual dimension took shape, until then underestimated. All this has been linked by students to their need for closeness with others and sharing the negative feelings caused by the pandemic, since forced isolation made them feel isolated in the moment of most intense fear. Therefore, they identified themselves with those who in hospitals could not be in contact with their loved ones. The course has therefore allowed students to discover the importance of the funeral rite and spirituality, allowing some to reconnect and explore their inner dimension regardless of their attitude toward religion (9). Other studies in literature have indeed highlighted how essential healthcare students believe receiving proper training also concerning spiritual aspects in patients' care is, and on the contrary the current lack of information and reflection upon these themes in healthcare University courses (40–42).

Lastly, the last thematic area concerned indeed the importance of death education courses for University students who will pursue careers in healthcare in the future. The issues that comprised this theme were the acquisition of awareness and personal reflection that allow recognition and control of the negative emotions that death and dying arouse; consequently, they also allow people to focus attention on life. Through the dissolution of taboos related to these topics, they have become aware and learned to recognize the negative emotions that occur in situations related to death. Finally, the positivity of the course was substantially derived from its focus on life and on the recognition of its importance. In general, all participants recognized the need for adequate preparation regarding the topics covered by the course, not only to live better and deal with these issues more serenely, but also for their future as health professionals. All participants therefore expressed only positive psychological and educational effects, valuing how much the course had allowed them to better manage the social trauma caused by COVID-19, and this appears to be in line with other literature, and especially studies involving also other healthcare students as well, which have confirmed how important it is to implement death education pathways for University students, especially for those studying to become health professionals (43, 44), as the present results highlighted as well.

## Conclusions

The present study highlighted how the death education course, conducted during the first phase of the COVID-19 pandemic in Italy, helped students become more aware of the issues addressed through contents and dialogues that encouraged participants to develop their own critical thinking. It allowed them to rediscover the value of reflection on the sense of limit, finitude of life, and the different representations of death. Since these meanings are usually taken for granted despite a general lack of awareness about the representations of death, the possibility to freely reflect upon and discuss these issues offered by the course rendered it therefore a significant tool to support participants during the confusing and highly dramatic period of the pandemic. Related to this, the research results showed also that the course helped participants consider the religious and spiritual aspect corresponding to the distal defenses indicated by TMT (4). Indeed, in line with the TMT perspective, religious and spiritual vision mitigates the concerns that arise from the awareness of death and allows people to focus their attention on everyday life without being assailed by death anxiety. Another fundamental element that has emerged from the research has been the rediscovered importance of funeral rites and to maintain a sense of community especially while facing extremely painful historical periods significantly characterized by grief. The study highlighted indeed a concrete need to address these problems and to link them to wider existential issues, such as the ability to improve reflection on the meaning of life and address the sense of limit. These results confirm once again the effectiveness of reflection on death and dying in critical periods, such as the COVID-19 pandemic, because it permits awareness of unconscious aspects that characterize the terror of death and gives them cultural and relative dimensions. This is especially important for healthcare professionals, who already find it generally difficult and rather distressing to manage the end of life of a patient due to lack of proper training on these aspects, and who therefore have to face, in this pandemic period, the additional psychological burden of dealing with even higher mortality salience and lack of proper personal and professional tools to elaborate it and break the silence and taboo that generally surrounds the experience of death, even for them. Considering this, the COVID-19 pandemic, its extremely high number of deaths and their massive burden for healthcare professionals who actively deal with them on a daily basis, represent a significant public health challenge to which more and more attention should be given in the near future. Given the current study findings therefore, the introduction and increase of death education pathways in University courses could be of great importance especially in the present situation of COVID-19 pandemic. They could indeed help young adults obtain the proper resources and personal skills they need to adequately face this period of intense uncertainty and mortality salience, and any other future challenge there might be in store, especially for healthcare students like psychologists for example, who will need to be able to properly address these themes with their patients in the near future. Proper Death Education courses should therefore be implemented keeping in mind the need to allow students to openly discuss, without prejudices or fear, the theme of death and human vulnerability, and express the related emotions through any mean they feel could be helpful.

## Limits and Future Perspectives

The research reports encouraging results that demonstrate the positive effects of death education; however, some limitations should be taken into account, including the inability to generalize the results given the specificity of conditions. Concerning this, future research should focus on significantly broaden the number of participants. In addition to this, the idea of a longitudinal study can be considered to see if the approach and discussion of the existential issues addressed can actually be changed and kept stable over time. Moreover, since the research participants were mostly all female, even though psychology students are usually more female than male future research could explore more precisely more male psychology students' point of view, comparing it to the one of female students. Other interesting aspects that could be investigated in future studies could also be the perspective of medical, nursing and social services students, as well as being conducted in other cultural contexts, since comparing psychology students' perspectives to the point of view of other healthcare and social services professionals could be particularly interesting and informative.

## Data Availability Statement

The raw data supporting the conclusions of this article will be made available by the authors, without undue reservation.

## Ethics Statement

The studies involving human participants were reviewed and approved by Ethical Committee for the Psychological Research of the University of Padua N 57BC2002FDF5CBD4292F5F86AA077F23. The patients/participants provided their written informed consent to participate in this study.

## Author Contributions

IT contributed to the research design and project planning, supervision of the research, analysis of the texts, methodology, and article writing. EI contributed to the project planning, analysis of the texts, and article writing. CC contributed to the interviews and analysis of the texts. LD contributed to the supervision, cooperation, and organization of the teamwork, and writing. SP contributed to the analysis of the texts, cooperation, and organization of the team work. HO and MW contributed to the writing. All authors contributed to the article and approved the submitted version.

## Conflict of Interest

The authors declare that the research was conducted in the absence of any commercial or financial relationships that could be construed as a potential conflict of interest.
